# Applications of Bacterial Degrons and Degraders — Toward Targeted Protein Degradation in Bacteria

**DOI:** 10.3389/fmolb.2021.669762

**Published:** 2021-05-07

**Authors:** Matylda Anna Izert, Maria Magdalena Klimecka, Maria Wiktoria Górna

**Affiliations:** Structural Biology Group, Biological and Chemical Research Centre, Department of Chemistry, University of Warsaw, Warsaw, Poland

**Keywords:** degron, degradation signal, degrader, targeted protein degradation, bacterial protease, proteolysis-targeting chimeras, induced degradation

## Abstract

A repertoire of proteolysis-targeting signals known as degrons is a necessary component of protein homeostasis in every living cell. In bacteria, degrons can be used in place of chemical genetics approaches to interrogate and control protein function. Here, we provide a comprehensive review of synthetic applications of degrons in targeted proteolysis in bacteria. We describe recent advances ranging from large screens employing tunable degradation systems and orthogonal degrons, to sophisticated tools and sensors for imaging. Based on the success of proteolysis-targeting chimeras as an emerging paradigm in cancer drug discovery, we discuss perspectives on using bacterial degraders for studying protein function and as novel antimicrobials.

## Introduction

Proteins in living cells undergo a constant process of synthesis and degradation. Protein degradation helps to maintain protein homeostasis by eliminating toxic aberrant proteins or regulating the levels of proteins needed under the given environmental conditions. The protein half-lives in *Escherichia coli* exist over a range of a few days down to a few minutes (Nagar et al., 2021). Bacteria, as unicellular organisms, are particularly exposed to severe environmental fluctuations including variations in temperature, nutrient availability, or the presence of toxic compounds (Pine, 1973; Mogk et al., 2011). Regulation of protein levels by degradation acts as one of the fastest ways to remodel the expressed proteome and enables rapid responses to these changing environmental conditions. As a result of stress, damage or a series of stochastic events, proteins may also unfold and aggregate (Mogk et al., 2011; Schramm et al., 2019). Such proteins can undergo either refolding or degradation since the loss of their structure leads to loss of function and aggregation of proteins may lead to cell death (Mogk et al., 2011; Schramm et al., 2019). Altered protein levels may be sensed by various feedback loops, involving transcriptional or translational regulators, which activate stress response pathways that help bacteria to quickly adapt to unfavorable conditions. Regulation of protein degradation pathways is well-conserved in all domains of life (Maurizi, 1992; Maurizi et al., 1994; Sauer and Baker, 2011; Miller and Enemark, 2016; Becker and Darwin, 2017; Varshavsky, 2017; Mahmoud and Chien, 2018). It is typically guided by the recognition of specific markers by cognate proteolytic complexes. The specific signals which turn the protein susceptible to degradation are called degrons (Varshavsky, 1991). Their size may vary from single amino acids, to short peptides, to post-translational modifications including tagging with a small protein (Luh et al., 2020).

Degrons have been used extensively in research as tools for manipulating protein levels, and here we describe the various applications and experimental designs exploiting bacterial degradation systems. In eukaryotes, the use of degrons has progressed beyond the laboratory and has engendered a new drug discovery field named Targeted Protein Degradation (TPD), based on induced proteasomal degradation of target proteins (Verma et al., 2020). This approach is a promising therapeutic strategy applied intensively in cancer research (Mullard, 2021), yet due to the lack of direct bacterial equivalents, it has not yet been applied in bacteria. We believe that exploiting degrons for induced degradation of endogenous target proteins could similarly empower chemical genetics approaches in bacteria and constitute an alternative to conventional antimicrobial drugs. This review focuses on the existing applications of bacterial degradation signals in the context of introducing TPD in bacteria as an approach to proteome engineering and developing novel degron-based antimicrobials.

## Degradation Pathways and Signals in Bacteria

Misfolded or unfolded proteins may be subjected to refolding by chaperones or they can be degraded and replaced by newly synthesized proteins. Proteases not only rescue cells from proteotoxic stress, but they also regulate levels of the existing proteins, maintaining the equilibrium between production and degradation (Alber and Suter, 2019). As refolding and degradation require high energy expenditure, typically powered by ATP hydrolysis, these processes are conducted by proteins belonging to the AAA+ family (ATPase Associated with diverse cellular Activities) (Neuwald et al., 1999; Santra et al., 2017; Rotanova et al., 2019). Protein degradation in bacteria is performed by proteases such as Clp complexes, Lon or the bacterial 20S proteasome which contain AAA+ domains (Table 1; Sauer and Baker, 2011). Typically, proteolytic complexes comprise an ATPase which unfolds polypeptide chains and a protease responsible for hydrolysis of peptide bonds. Bacteria also have many other proteases which carry out various specific functions in different intra- or extracellular localizations. In this review we focus on the family of AAA+ proteases since they are well-characterized, ATP-powered, highly processive, have a broad range of substrates and are primarily located in the cytoplasm, which—like the eukaryotic proteasome—makes them good candidates for TPD. Two of the most ubiquitous proteases, serine proteases ClpP and Lon, might be the most promising choice for designing a targeted degradation system which could be applied to a broad range of bacterial pathogens with minor modifications.

**TABLE 1:** A list of bacterial proteases with examples of their substrates.

Protease	ATPase partner	Adaptor or regulator	Substrates	Degron location	Sequence	References
ClpP	ClpX (*E. coli*)	SspB	ssrA-tagged proteins	C-terminus	AANDENYALAA	Gottesman et al., 1998
			RseA^1–108^ (cleaved)	C-terminus	VRPWAAQLTQMGVAA	Flynn et al., 2004
		–	MuA	C-terminus	RRKKAI	Harshey et al., 1985; Levchenko et al., 1995
		–	FtsZ	C-terminus	AKEPDYLDIPAFLRKQAD	Camberg et al., 2009
		RssB	RpoS (σS)	N-terminus	KVHDLNEDAEFDENGVE VFDEKALVEQEP	Stüdemann et al., 2003
		–	λO	N-terminus	TNTAKILNFGR	Flynn et al., 2003
		–	Dps	N-terminus	STAKLVKSKAT	Flynn et al., 2003
		–	OmpA	N-terminus	MKKTAAIAIAV	Flynn et al., 2003
	ClpX (*B. subtilis*)	–	Poly-Ala-tagged proteins	C-terminus	Poly-Ala	Lytvynenko et al., 2019
		YjbH	Spx	C-terminus	FLPRKVRSFQLRE	Awad et al., 2019
		CmpA	SpoIVA	n.d.	n.d.	Tan et al., 2015
	ClpX (*C. crescentus*)	CpdR/RcdA/PopA	CtrA	N-terminus	DPNEQVNAA	Domian et al., 1997; Joshi et al., 2015
		CpdR/RcdA	TacA	C-terminus	TLEEIERDLIQH	Joshi et al., 2015
		CpdR	PdeA	C-terminus	GAAPVKARG	Rood et al., 2012
		SocA	SocB	n.d.	n.d.	Aakre et al., 2013
	ClpA (*E. coli*)	ClpS	N-degron pathway	N-terminus	L, F, W, Y	Dougan et al., 2002; Ninnis et al., 2009; Schuenemann et al., 2009
	ClpC (*B. subtilis*)	MecA	ComK	C-terminus	FMLYPKEERTMIYD FILRELGERY	Prepiak and Dubnau, 2007
			ComS	N-terminus	IILYPR	Ogura et al., 1999; Prepiak and Dubnau, 2007
		McsB	CtsR	Internal (tagged Arg)	pArg	Trentini et al., 2016
			MgsR	Internal (tagged Arg)	pArg	Lilge et al., 2020
Lon	Lon AAA+ domain	–	RcsA (*E. coli*)	n.d.	n.d.	Stout et al., 1991; Gur and Sauer, 2009
		–	SulA (*E. coli*)	C-terminus	ASSHATRQLSGLKIHSNLYH	Ishii et al., 2000; Gur and Sauer, 2009
		–	Y2853 (*Y. pestis*)	C-terminus	PLTATSYPIIH	Puri and Karzai, 2017
		–	UmuD (*E. coli*)	N-terminus	FPLFSDLVQCGFPSP	Gonzalez et al., 1998
		–	ZntR (*E. coli*)	N-terminus	n.d.	Pruteanu et al., 2007
		–	Unfolded proteins	Internal	Hydrophobic amino acids	Gur and Sauer, 2008b
		–	DnaA (*C. crescentus*)	N-terminus	MSLSLWQQCLARL QDELPATEF	Liu et al., 2019
		–	SoxS (*E. coli*)	N-terminus	SHQKIIQDLIAWIDEHIDQ	Shah and Wolf, 2006
		HspQ	YmoA (*Y. pestis*)	n.d.	n.d.	Puri and Karzai, 2017
		–	PerR (*B. subtilis*)	Internal, oxidation	NNLRVFR	Ahn and Baker, 2016
FtsH (*E. coli)*	FtsH AAA+ domain	n.d.	LpxC	C-terminus	LAFKAPSAVLA	Führer et al., 2006
		λCIII	λCII	C-terminus	RSEQIQMEF	Kobiler et al., 2002
		n.d.	RpoH (σ^32^)	n.d.	n.d.	Herman et al., 1995, 2003
		–	YfgM	N-terminus	EIYENENDQVEAV	Bittner et al., 2015
		–	YccA	N-terminus	VSSSHDRT	Kihara et al., 1999
		–	SecY	n.d.	n.d.	Kihara et al., 1995
HslV (ClpQ) (*E. coli*)	HslU (ClpY)	–	RcsA	n.d.	n.d.	Chang et al., 2016
		–	SulA	Internal	GFIMRP	Chang et al., 2019
		–	YbaB	n.d.	n.d.	Tsai et al., 2017
		–	RpoH (σ^32^)	n.d.	n.d.	Kanemori et al., 1997
20S Proteasome (*M. tuberculosis*)	Mpa	–	Pup-tagged proteins	Internal (tagged Lys)	MAQEQTKRGGGGGDD DDIAGSTAAGQERREKLTE ETDDLLDEIDDVLEENAE DFVRAYVQKGGQ	Cole et al., 1998
	–	Bpa	Unstructured proteins	Internal	Hydrophobic amino acids	Delley et al., 2014
	Cpa	–	n.d.	n.d.	n.d.	Ziemski et al., 2018

**n.d., not determined.**

### Proteolytic Complexes Based on ClpP and Lon

**FIGURE 1:**
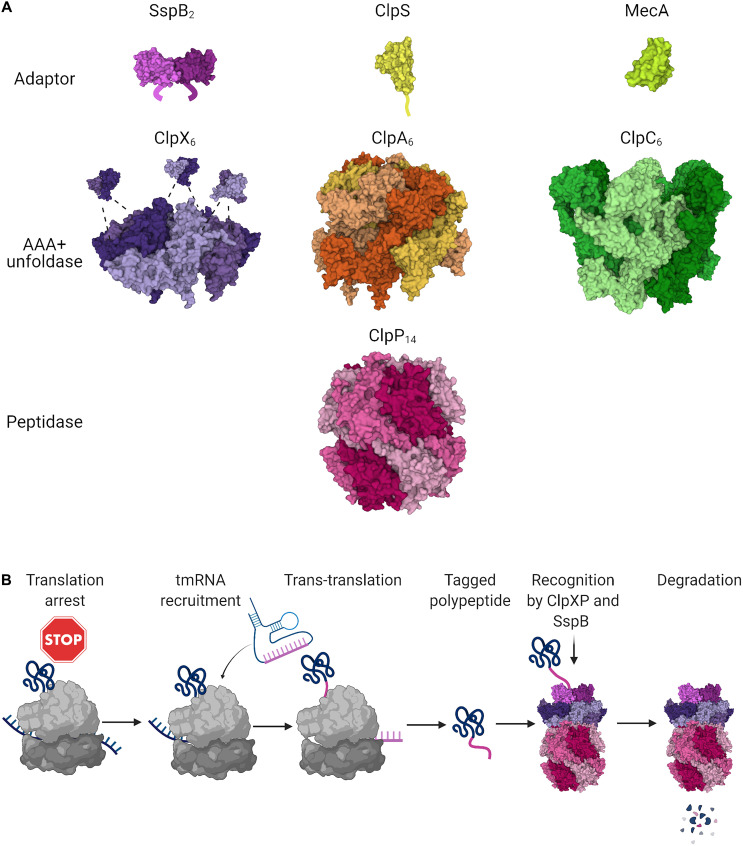
ClpP-based proteolytic systems in bacteria. **(A)** The tetradecameric peptidase ClpP (PDB ID 6NB1; Mabanglo et al., 2019) can be assisted in substrate unfolding and recognition by the hexameric unfoldases ClpX (PDB ID 6PP5; Fei et al., 2020), ClpA (PDB ID 6UQO; Lopez et al., 2020), or ClpC (PDB ID 3J3S; Liu et al., 2013) from the AAA+ family. The unfoldases bind to one or both faces of the ClpP double barrel, promoting its opening. Each unfoldase can cooperate in substrate selection with its cognate adaptor proteins: the C-terminal XB tail of the dimeric SspB is bound by the Zinc Binding Domain of ClpX (PDB ID 2DS7; Park et al., 2007), the N-terminal extension of ClpS (PDB ID 3O1F; Román-Hernández et al., 2011) baits ClpA, while MecA (PDB ID 3J3S; Liu et al., 2013) cooperates in *B. subtilis* with ClpC. **(B)** The highly conserved tmRNA system rescues stalled ribosomes and appends ssrA degrons through trans-translation. tmRNA provides the coding template for the ssrA peptide which contains an SspB-binding motif and C-terminal residues bound by ClpX. The ribosome rescue event results in the production of a fusion protein with the C-terminally appended ssrA degron which in *E. coli* is targeted for degradation primarily through the SspB-ClpXP pathway. Figures were created with BioRender.com and Mol* (Sehnal et al., 2018).

The gene encoding the caseinolytic protease ClpP was found in most of the bacterial genomes with the exception of Mollicutes (Yu and Houry, 2007). ClpP also exists in eukaryotes, mostly in organelles such as chloroplasts and mitochondria (Yu and Houry, 2007). It is an ATP-dependent serine protease, which associates with AAA+ chaperones (Figure 1A). ClpP oligomerizes into a tetradecameric barrel-like structure composed of two stacked heptameric rings (Wang et al., 1997). In some bacteria with two paralogous genes *clpP1* and *clpP2* (such as *Mycobacteriaceae*, *Listeriaceae*, *Pseudomonaceae*), ClpP1 and ClpP2 each form homoheptameric rings which stack on top of each other. Each barrel possesses 14 active sites facing the inside of the central channel (Wang et al., 1997). Because of the small diameter of the entrance pore, ClpP by itself can degrade only unstructured proteins and short peptides (Thompson and Maurizi, 1994). In order to degrade larger proteins, ClpP has to cooperate with AAA+ chaperones which unfold substrates.

The ClpP partner unfoldases ClpX, ClpA, and ClpC have a typical structure for AAA+ proteins with a characteristic α/β fold, Walker A and B motifs which mediate ATP binding and hydrolysis, and C-terminal helical bundle (Miller and Enemark, 2016). They form homohexameric rings which bind to one or both faces of the ClpP barrel. It is the docking of highly conserved Ile-Gly-Phe or Ile-Gly-Leu (IGF/IGL) loops of the unfoldases in the hydrophobic pockets of ClpP that causes opening of the ClpP central pore and enables degradation of larger peptides (Lee et al., 2010; Alexopoulos et al., 2012). ClpX has one ATPase domain while ClpA and ClpC have two of them (Sauer and Baker, 2011). ClpX is the best conserved ClpP partner and is found in most bacteria. ClpA and ClpC are present in general, respectively, in Gram-negative or Gram-positive bacteria. Some proteobacteria were found to have both genes, although they are functionally redundant and could be a result of horizontal gene transfer (Miller et al., 2018). Certain proteases such as Lon do not need to form a complex with an unfoldase, since they comprise both proteolytic and ATPase domains and therefore have chaperone activity themselves (Sauer and Baker, 2011).

In general, the processive protease subunits are not highly specific, so that substrate engagement is usually mediated by degrons which are recognized by the AAA+ subunits. Degrons might interact directly with unfoldases or with adaptor proteins which help in delivering the substrates to the proteolytic complexes (Kuhlmann and Chien, 2017; Mahmoud and Chien, 2018) (summarized in Table 1). Degradation of certain proteins requires multiple adaptors acting in concert (Joshi et al., 2015). Adaptors can enhance the action of the protease complex by improving the affinity of the AAA+ protein for the substrate (Wah et al., 2002; Román-Hernández et al., 2011), pulling the substrate to facilitate engagement by the proteolytic complex (Rivera-Rivera et al., 2014), or enabling the assembly of the ATPase hexamers (Kirstein et al., 2006). The presence of adaptors can also reprogram the protease complex by inhibiting the degradation of other protease substrates (Dougan et al., 2002; Torres-Delgado et al., 2020) or preventing autodegradation of the unfoldase (Dougan et al., 2002). Not all proteases and substrates require an adaptor, for instance there are few known examples of proteins activating or reprogramming Lon (Puri and Karzai, 2017) and this protease can exert most of its functions without the aid of accessory proteins. Conversely, ClpCP requires an adaptor protein or substrate for ClpC complex formation (Kirstein et al., 2006; Trentini et al., 2016) and chaperone activity (Schlothauer et al., 2003; Trentini et al., 2016). One such example of ClpC adaptor in *Bacillus subtilis* is MecA, which is degraded together with the proteolytic substrates instead of being recycled and the protease complex is being disassembled upon completing degradation (Schlothauer et al., 2003; Mei et al., 2009).

### C-Degrons Appended Through Trans-Translation

Bacterial ribosome rescue and degradation of nascent proteins stalled on ribosomes requires a process called trans-translation. Upon translation arrest in bacteria, a tmRNA molecule is recruited, the translated mRNA is cleaved and it dissociates from the ribosome (Janssen and Hayes, 2012). The translation resumes on the tmRNA template and a short peptide called ssrA is appended to the synthesized polypeptide. The ssrA tag is a C-terminal degradation signal (C-degron) and the tagged protein is eliminated predominantly by the ClpXP complex (Figure 1B; Keiler, 2008). Trans-translation seems to be a highly significant quality control mechanism since genes encoding tmRNA and proteins involved in this process are highly conserved in bacteria and ssrA mutants show growth and virulence defects (Oh and Apirion, 1991; Keiler, 2008). The ssrA tagging is not only a rescue mechanism but it is also involved in the regulated proteolysis of certain substrates (Hong et al., 2007). Degradation of ssrA-tagged substrates is facilitated by the stringent starvation protein SspB (Wah et al., 2002; Dougan et al., 2003; Farrell et al., 2005). This protein acts as an adaptor binding to the zinc-binding domain of ClpX and delivering the tagged proteins to the proteolytic complex (Dougan et al., 2003; Wojtyra et al., 2003; Park et al., 2007). However, SspB is not indispensable for degradation of ssrA tagged proteins by ClpXP and it was found only in certain proteobacteria such as *E. coli* and *Caulobacter crescentus* (Lessner et al., 2007; Chowdhury et al., 2010). Though ClpXP is the main proteolytic complex responsible for eliminating products of trans-translation, the ssrA-tagged proteins can also be degraded by ClpAP, Lon, or FtsH proteases (Gottesman et al., 1998; Farrell et al., 2005; Gur and Sauer, 2008b; Hari and Sauer, 2016).

Recently, an alternative ribosome quality control pathway was discovered in *Bacillus subtilis*. The mechanism is based on the recognition of C-terminal poly-Ala tails by ClpXP (Lytvynenko et al., 2019). A similar system exists in yeast, where Rcq2 protein adds C-terminal Ala-Thr tails (CAT-tails) to the polypeptides stalled on ribosomes and promotes their ubiquitination and degradation (Yonashiro et al., 2016; Kostova et al., 2017). In bacteria, Rcq2 homolog (RqcH) together with Hsp15/RqcP recruit Ala-tRNA to the stalled peptides which are then degraded in a ClpXP-dependent manner (Lytvynenko et al., 2019; Crowe-McAuliffe et al., 2020; Filbeck et al., 2020). This degradation pathway also exists in a number of Gram-positive bacteria and Archea which suggests that it was formed during the early evolution of life (Lytvynenko et al., 2019).

### N-Degron Pathway

The composition of the N-terminus was found to regulate the stability of proteins and therefore determine their half-lives. The N-degron pathway was identified in bacteria as well as in yeast and higher eukaryotes, although the destabilizing amino acids vary between the organisms (Tobias et al., 1991; Dougan et al., 2010, 2012; Varshavsky, 2019). In bacteria the primary destabilizing residues are hydrophobic and aromatic amino acids such as Leu, Phe, Trp, and Tyr (Tobias et al., 1991; Ninnis et al., 2009; Schuenemann et al., 2009; Varshavsky, 2011) while secondary destabilizing residues could be Met or the charged amino acids Asp, Glu, Lys, Arg (Tobias et al., 1991; Graciet et al., 2006; Ninnis et al., 2009; Varshavsky, 2011; Dougan et al., 2012). Typically, bacterial N-degrons are formed either by endoproteolytic processing or attachment of a primary destabilizing residue by an amino acid transferase to specific N-terminal residues (Tobias et al., 1991; Ninnis et al., 2009; Dougan et al., 2010; Humbard et al., 2013). Some studies suggest that formylated N-terminal Met can serve as a degradation signal (Piatkov et al., 2015). In eukaryotes, another way of generating N-degrons involves exposure of destabilizing residues by removal of the N-terminal Met (Varshavsky, 2019), although this is yet to be demonstrated in bacteria. The canonical example of the N-degron pathway in Gram-negative bacteria involves the ClpAP complex (Tobias et al., 1991) and the ClpS adaptor which is also referred to as an N-recognin (Erbse et al., 2006; Schmidt et al., 2009; Schuenemann et al., 2009). The N-terminal amino acids of the substrate are bound by the core of ClpS (Wang et al., 2008; Rivera-Rivera et al., 2014). The ClpS N-terminal Extension (NTE) fragment enters the central channel of the protease complex and releases the substrate which is then unfolded and degraded by ClpAP, while ClpS is being recycled (Román-Hernández et al., 2011; Rivera-Rivera et al., 2014). The presence of ClpS significantly reduces the affinity of ClpAP to ssrA-tagged proteins suggesting that it has a complex mode of action, delivering the N-end rule proteins while preventing degradation of other ClpAP substrates (Dougan et al., 2002; Torres-Delgado et al., 2020). No sequelogs of ClpS were identified in Gram-positive bacteria or Archea suggesting that this degradation pathway occurs only in Gram-negative bacteria and eukaryotes (Varshavsky, 2011).

### Constitutive and Conditional Degrons

Degrons naturally occurring in protein sequences are also a part of natural regulation of protein half-lives. Their timely recognition and degradation helps to maintain proteostasis and regulate various cellular processes (Stüdemann et al., 2003; Camberg et al., 2009; Bhat et al., 2013; Buczek et al., 2016; Arends et al., 2018). Proteases may recognize a pool of protein sequences. The C-terminal motifs identified in ClpXP substrates are similar to the ssrA tag or the MuA transposase C-terminal sequence and the N-terminal motifs have high homology with the N-terminus of the outer membrane protein OmpA or λO phage replication protein (Flynn et al., 2003). Bacterial proteases are responsible for removal of prematurely terminated or unfolded proteins (Gur and Sauer, 2008b; Van Melderen and Aertsen, 2009; Sauer and Baker, 2011; Arends et al., 2018; Mahmoud and Chien, 2018). Their degradation is mediated by recognition of regions with aromatic amino acid side chains and the absence of small polar amino acids which can be exposed upon unfolding (Gur and Sauer, 2008b; Van Melderen and Aertsen, 2009). As an example, an unstructured N-terminal fragment of β-galactosidase constitutes a degradation signal for the Lon protease, even though the full length folded protein is not degraded by Lon (Gur and Sauer, 2008b). Degron exposure under extreme conditions is often a part of the stress response. Cryptic degrons may become accessible upon temperature stress, oxidative environment or endoproteolytic cleavage of the substrate protein (Sauer and Baker, 2011). Regulated degradation is also mediated by other bacterial proteases activated by heat shock including HslUV (Baytshtok et al., 2021) or FtsH which can degrade both cytoplasmic and membrane proteins (Bittner et al., 2015, 2017).

### Post-translational Modifications Directing Proteins for Degradation

Marking proteins for degradation is also mediated by post-translational modifications such as phosphorylation or attachment of a small protein. Arginine phosphorylation by protein-arginine kinase McsB is a degradation signal for ClpCP in *Bacillus subtilis* (Kirstein et al., 2007; Elsholz et al., 2011, 2012; Trentini et al., 2016). Degradation of phosphorylated proteins seems to be involved in adaptation to high temperatures (Trentini et al., 2016). Interestingly the presence of phosphorylated substrates promotes formation of ClpCP complex and enables degradation even in absence of adaptors (Trentini et al., 2016).

The post-translational modification which targets proteins to the 20S proteasome present in some bacterial orders (Nitrospirales and Actinomycetales) resembles the one in eukaryotes (Striebel et al., 2009; Jastrab and Darwin, 2015; Fuchs et al., 2018; Becker et al., 2019; Müller and Weber-Ban, 2019). Eukaryotic proteins are targeted for the proteasome by conjugation of ubiquitin by the cascade action of enzymes E1, E2, and E3 (Varshavsky, 2017). Ubiquitinated proteins are recognized and degraded by the proteasome. Analogously, in actinobacteria, proteins directed for degradation are tagged on lysine side chains by a small prokaryotic ubiquitin-like protein (Pup). Pup is attached covalently by the single action of Pup protein ligase PafA (Pearce et al., 2008). It can be removed by Dop (Pup deaminase/depupylase) which not only recycles Pup and regulates degradation rates (Pearce et al., 2008; Burns et al., 2010; Imkamp et al., 2010) but also activates Pup (Striebel et al., 2009; Elharar et al., 2017). Pup is partially disordered and remains disordered upon binding to the target proteins (Chen et al., 2009; Liao et al., 2009; Barandun et al., 2017). This might contribute to protein degradation, since degrons which target substrates to the proteasome and other proteases are often unstructured peptides (Prakash et al., 2004; Gur and Sauer, 2008b; Kim et al., 2011; Hughes et al., 2018; Inobe et al., 2018). Despite certain similarities, bacterial Pup tagging is simpler than the eukaryotic ubiquitin-proteasome system and the differences between them suggest that they developed independently (Imkamp et al., 2015). Pup-tagged proteins are recognized and bound by Mpa, an AAA+ unfoldase which is an activator protein for the bacterial proteasome (Darwin et al., 2004; Wu et al., 2017), competing for 20S binding with two other regulators Bpa (recognizing unstructured proteins) (Delley et al., 2014) and Cpa (Ziemski et al., 2018). Since the Pup-proteasome degradation system is only found in Actinobacteria but is lacking in other bacterial phyla, it has a limited potential as a universal proteolytic machinery in targeted degradation. However, since it plays a significant role in a number of important pathogens such as *Mycobacterium tuberculosis* (Darwin et al., 2003; Gandotra et al., 2007), it might be exploited for fighting antimicrobial resistant mycobacteria which cause tuberculosis.

## Tools for Protein Degradation and Their Applications

Two strategies find use in targeting proteins for degradation: fusing proteins with degrons or applying degrader molecules. We describe these two approaches in turn and how they may be used to modify protein stability for various applications in functional studies of proteins, synthetic biology or drug discovery.

### Applications of Bacterial Degrons

Studies of protein function often exploit fusion constructs appending otherwise stable proteins with degrons to enable tight regulation of protein levels. Since the ssrA-tagging system is the most extensively studied, ssrA is currently the only degron widely used for modification of protein stability in bacteria (Fritze et al., 2020). Given the high efficiency of degradation and the precision of control conferred by adaptor proteins, using degrons can serve as a diverse tool for reverse genetics and clever synthetic biology applications. However, since a single degron can be recognized by multiple proteases under natural conditions, engineered proteins with attached degrons may be susceptible to degradation by several pathways, which can make degradation control more difficult (Ogle and Mather, 2016; Butzin and Mather, 2018). To increase degradation specificity and stringent control, different strategies may be applied, such as using heterologous degrons recognized by degradation systems from other organisms; other approaches include split-adaptor systems or degrons which are exposed upon specific proteolytic cleavage.

#### Homologous Use of Fine-Tuned Degron Variants

Degron-induced protein degradation is one of the ways of regulating gene expression in loss-of-function protein studies. A collection of bacterial expression-regulating elements including different constitutive promoters, ribosome-binding sites and degrons enabled modifications in *B. subtilis* on multiple levels: transcription, translation and protein stability (Guiziou et al., 2016). The proteolysis rate of the target protein could be regulated by the addition of a ssrA variant. In total, 10 different versions of ssrA with a modified tripeptide at the C-terminus were used to tune the protein levels constituting a valuable tool for protein research.

Precise regulation of protein expression is valuable in many synthetic biology applications. Addition of degrons to proteins involved in synthetic circuits can prevent protein accumulation and therefore enable fast response to the changing concentrations of inducers and repressors. Degrons are widely used in the design of genetic oscillators which periodically switch from one state to another *in vitro* or *in vivo* (Stricker et al., 2008; Purcell et al., 2010; Niederholtmeyer et al., 2015; Potvin-Trottier et al., 2016). Stringent regulation of a protein half-life can also be applied in more sophisticated synthetic circuits, for example a digital data storage platform in *E. coli* capable of recording cellular events using fluorescent reporters (Bonnet et al., 2012). Adding a ssrA tag to the components driving DNA recombination helped to create a resettable system which holds its state for multiple generations of cells.

#### Heterologous Use of Degrons

Because of the high conservation of tmRNA tagging system, ssrA tags can be introduced to different species of bacteria and still be recognized and processed by their cognate endogenous or transgenic proteases. Interspecies differences such as dependence of degradation on adaptors can be used to ensure stringent control of protein degradation and the diversity in recognition of degrons can be exploited to avoid interference with endogenous degradation systems.

Involvement of SspB is not necessary for the degradation of ssrA-tagged proteins, and not all bacteria express homologues of this ClpXP adaptor (McGinness et al., 2006). The absence of SspB homologues in *Bacillus subtilis* and mycobacteria was exploited to create two similar systems based on the ssrA derived degrons and inducible expression of SspB (Griffith and Grossman, 2008; Kim et al., 2011). An ssrA tag variant featuring Asp-Ala-Ser at the C-terminus and four residues inserted between the ClpX and SspB binding sites (referred to as DAS+4 tag) was used in both cases (McGinness et al., 2006; Griffith and Grossman, 2008; Kim et al., 2011). Such degrons cause rapid protein degradation in the presence of SspB, while they are stable when the adaptor is absent (McGinness et al., 2006). In *Bacillus subtilis* this degron was mutated and optimized for enhanced stability and SspB dependence. This enabled rapid ClpX-dependent degradation of tagged proteins strictly upon induction of SspB expression. The system was applied for inducible degradation of ComA transcriptional regulator and several proteins involved in sporulation (Griffith and Grossman, 2008). However, in such an approach the different degradation tags and the different variants of the promoter controlling SspB expression may need to be tested for the optimal degradation control of each individual protein. *C. crescentus* SspB and the degron optimized for this adaptor were used in parallel to *E. coli* degradation components to show that the system can be modified for more complex applications enabling orthogonal regulation of degradation of two proteins simultaneously (Griffith and Grossman, 2008).

Similarily, a DAS+4 tag was introduced at the C-terminus of some reporter proteins in *M. smegmatis* and *M. tuberculosis*. Transfection of mycobacteria with an SspB-encoding plasmid with an inducible promoter enabled regulation of the levels of the target proteins. This system was also tested on the endogenous RNA polymerase subunit β (RpoB). Attachment of the DAS+4 tag led to inactivation of RNA polymerase and caused growth inhibition. This supports the applicability of degron tagging for identification of novel drug targets while omitting limitations of transcriptional gene silencing which can be lengthy and inefficient (Kim et al., 2011).

**FIGURE 2:**
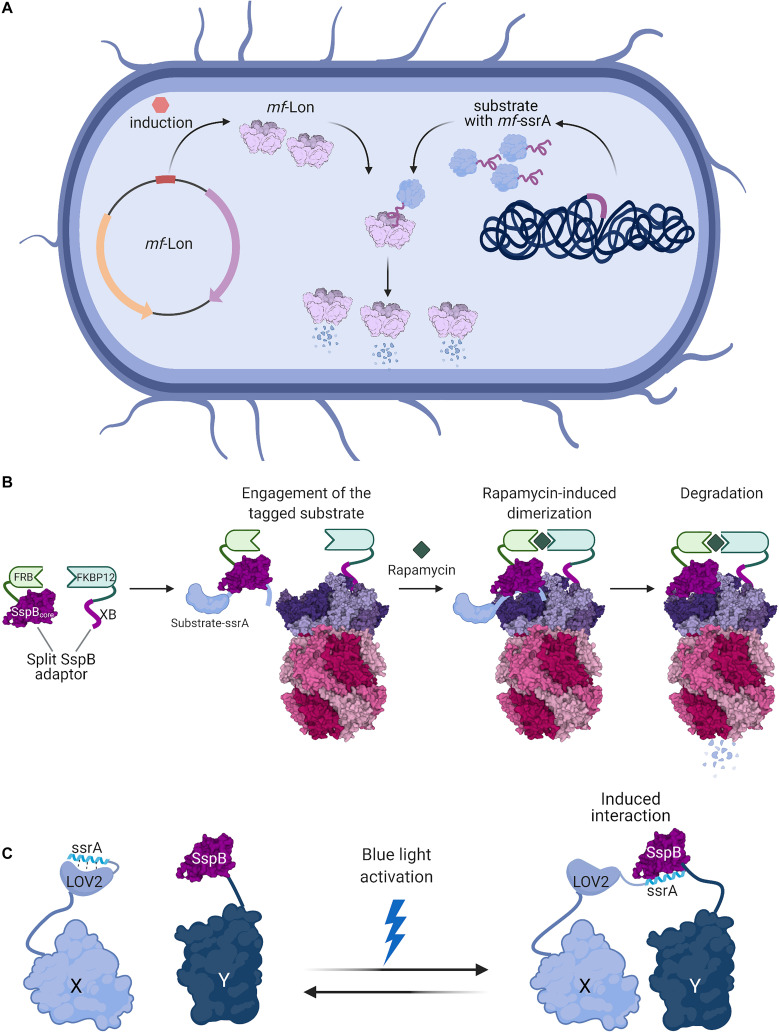
Bacterial degrons are used as tools for controlled protein degradation and interaction modules. **(A)** Induction of heterologous expression of *M. florum* Lon (PDB ID 1RRE; Botos et al., 2004) protease in *E. coli* or *C. crescentus* cells enables selective degradation of proteins fused with *mf*-ssrA degrons (Cameron and Collins, 2014). **(B)** A split-adaptor system can be used to specifically control the degradation of homologously expressed proteins. Protein constructs encoding the SspB core domain fused to FRB and the SspB C-terminal XB tail fused to FKBP12 can be made to interact by the addition of the small molecule rapamycin. The SspB core domain recognizes the substrate appended with a ssrA DAS+4 degron, while the SspB XB tail binds to ClpX. The rapamycin-induced assembly of this split-adaptor system results in the degradation of the target protein (Davis et al., 2011). **(C)** The degron-adaptor interaction can be used to co-localize proteins in a light-inducible manner. Protein X is fused to LOV2 with a C-terminally appended ssrA-derived sequence, while the second protein Y is fused to SspB. In the dark, ssrA is bound by LOV2 and precluded from interaction with SspB. Light-induced conformational changes in LOV2 cause the release of the ssrA degron, which is recognized by SspB and mediates the interaction between the proteins X and Y (Guntas et al., 2015). Figures were created with BioRender.com and Mol* (Sehnal et al., 2018).

A different degron-recognizing protease was employed by Cameron and Collins to create a modular system applicable in diverse bacterial species, based on the *Mesoplasma florum* ssrA-tag (Cameron and Collins, 2014). Mycoplasma have a minimal genome encoding only two members of the AAA+ protease family: FtsH and Lon. Despite having a significantly smaller number of genes, Mycoplasma retained the trans-translation system which indicates the importance of stalled ribosome rescue (Gur and Sauer, 2008a). However, *M. florum* ssrA differs in length and sequence from tmRNAs typically found in bacteria. Due to the lack of the ClpXP complex (the main protease eliminating ssrA-tagged proteins in most bacterial genera), degradation of ssrA-tagged substrates in *M. florum* is mediated by Lon (Gur and Sauer, 2008a). As *mf*-ssrA is not recognized well by Lon from other bacteria and *mf*-Lon does not efficiently degrade proteins with distinct tmRNA tags, introducing them into a different organism enabled the creation of an efficient inducible degradation system (Gur and Sauer, 2008a; Cameron and Collins, 2014). Inserting *mf*-ssrA-derived degradation tag at the C-terminus of the protein of interest and *mf*-Lon under a tetracyclin-inducible promoter, either on a plasmid or in the LacZ locus, provides a tool for regulated protein degradation (Figure 2A). The utility of this system was proven in *E. coli* as well as in *Lactococcus lactis*, suggesting it may be widely applicable in bacteria. In *E. coli*, a simple toggle switch circuit was engineered to show the utility of this system in synthetic biology. The *mf*-ssrA tag was further modified to improve protein stability in the absence of *mf*-Lon by reducing recognition by endogenous proteases (Lv et al., 2019). Finally, the Essential Protein Degradation library which is composed of 238 strains with tagged essential proteins and inducible expression of *mf*-Lon proved that such an artificial degradation system can be exploited in basic protein function research and in drug discovery screens (Cameron and Collins, 2014).

#### The Split-Adaptor System for Small-Molecule Induced Degradation

Rapid control of protein degradation can also be achieved by chemically induced dimerization of adaptor domains. This approach exploits the interaction between FRB (a domain of mTOR serine/threonine kinase) with FKBP12 (peptidyl-prolyl cis-trans isomerase) upon binding to rapamycin (Chen et al., 1995; Figure 2B). The core domain of the SspB adaptor protein and its ClpX-binding peptide were split and fused to FRB and FKBP12, respectively. Introducing these constructs in an *sspB*- strain allowed the induction of degradation of proteins tagged with a ssrA-DAS+4 degron. Additionally, the degradation could be easily switched off by removal of rapamycin (Davis et al., 2011).

#### Degrons Exposed by Protein Cleavage

Several systems for controlling protein degradation incorporate terminal degrons in internal sites. The degrons are protected by endopeptidase recognition peptides. The degradation can be induced by expression of transgenic endopeptidases such as TEV and HIV-2 or by conditions which cause self-cleavage of the protein. When the endopeptidase is not induced, the degron-tagged protein remains stable, but when the cleavage is induced, the protective sequence is removed, the degron is exposed, and the target undergoes degradation by either ClpXP or ClpAP complex. This is a widely applicable approach since it was used in different organisms for both N- and C-degrons (Wei et al., 2011; Sekar et al., 2016; Liu et al., 2017). A modified ssrA system was tested in *M. smegmatis* on different antibiotic targets, which in many cases resulted in increased susceptibility of the bacteria to antimicrobials, proving that regulated protein degradation can be a valuable tool in drug development (Wei et al., 2011).

Another system using an endoprotease recognition site and a ssrA degron was developed to enable growth-independent protein production. Functional engineering of ssrA/NIa-based flux control (FENIX) is based on a C-terminal fusion of NIa protease recognition site followed by the ssrA sequence (Durante-Rodríguez et al., 2018). Under normal conditions the protein of interest expressed under a constitutive promoter is continuously degraded, but upon induced expression of NIa protease the degron is cleaved off resulting in accumulation of a stable protein, such as the acetyl-CoA transferase (PhaA) which is involved in the synthesis of polyhydroxybutyrate (Steinbüchel et al., 1992; Durante-Rodríguez et al., 2018). This allowed the uncoupling of protein production from cell growth to manipulate the metabolic flux for more efficient biopolymer synthesis (Durante-Rodríguez et al., 2018). This is of particular importance for the production of proteins which interfere with bacterial growth or for the synthesis of toxic proteins and enzymes. The FENIX approach may have important implications for industrial production of enzymes and polymers in bacteria.

#### Extraction of Components From Macromolecular Complex

The high affinity between a degron and a specific protease can be exploited to separate the target protein from more complex structures. The pulling force created by ClpX is so strong that it can separate tagged proteins from the bacterial membrane and nucleic acid complexes (Burton and Baker, 2005; Chai et al., 2016; Abeywansha et al., 2018). The high affinity of ClpX to its substrates was used as “molecular tweezers” to extract a 50S subunit component, ribosomal protein L22 (Moore et al., 2008). L22 is crucial for correct ribosome assembly since it forms multiple stable contacts with 23S rRNA (Moore and Sauer, 2008). Investigations into the functions of this protein are limited since its genomic deletion disrupts complex formation and therefore affects the whole ribosome. In order to better understand the specific roles of L22, the endogenous L22 protein in a *clpX*- strain was exchanged for a version with an N-terminal His-tag and an unstructured titin domain, followed by ssrA at the C-terminus. *In vitro* degradation by ClpXP of tagged L22 in isolated ribosomes was not complete, the protease degraded only the titin ssrA part and leaving L22 protein intact. Partial destabilization of the ribosomal complex due to a reduced concentration of magnesium in the buffer allowed efficient L22 degradation, but did not result in disassembly of the whole ribosomal subunit. Because magnesium ions are involved in proper folding and interactions of rRNAs (Allen and Wong, 1986), moderate reduction of magnesium levels likely loosened the ribosome structure and therefore enabled the extraction. Even though the harsh extraction conditions resulted in a decreased translational activity of the isolated ribosomes, the ssrA-mediated degradation of proteins has a potential use in studying the functions of individual components of complex biological assemblies without disrupting their whole structure (Moore et al., 2008).

#### Acoustic Biosensors

An interesting example of degron use was creation of an acoustic biosensor by affecting gas vesicle properties. Multi-protein gas vesicles can be formed by mixing a small hydrophobic protein GvpA and a small hydrophilic protein GvpC (Walsby, 1994). These structures exist naturally in aquatic cyanobacteria and regulate their buoyancy and phototaxis (Walsby, 1994). The presence of gas vesicles was found to improve ultrasonic contrast and therefore constitutes a promising tool for molecular imaging (Yang et al., 2017). The vesicles can be modified by introducing a protease-recognized sequence in the GvpC protein which forms a scaffold on the vesicle surface. Upon protease cleavage the vesicles retain the same morphology, but their physical properties such as pressure resistance change, which affects the non-linear ultrasound contrast (Lakshmanan et al., 2020). In this way the protease activity can be tracked by monitoring the contrast change upon proteolytic cleavage. Tagging a gas vesicle protein with ssrA and introducing it in bacterial strains with ClpXP expression under the control of an inducible promoter allowed the monitoring of enzymatic activity in synthetic circuits. Moreover, engineering *E. coli* with ssrA-tagged gas vesicles controlled by an arabinose-induced ClpXP can also be used to improve ultrasound contrast in the gastrointestinal tract in infected mice (Lakshmanan et al., 2020).

#### Photoswitches Using Affinity Between ssrA and SspB

Degron-adaptor interactions can be also exploited for their high affinity as binding modules. The SspB-binding fragment of ssrA fused with a photoswitchable domain were used to create a light-inducible dimer (LID) with SspB (Lungu et al., 2012). LIDs are often based on photoactivatable proteins which naturally occur in plants. Upon exposure to blue light, the proteins change their conformation and expose their ligand-binding sites (Salomon et al., 2000; Harper et al., 2003). Fusing a fragment of the ssrA peptide to an AsLOV2 protein domain which undergoes structural rearrangement upon light exposure helped to create a system for precise control of protein interactions (Lungu et al., 2012; Guntas et al., 2015; Figure 2C). Under normal conditions, the ssrA fragment is embedded in the AsLOV2 protein and therefore unavailable for SspB binding, but upon light activation the AsLOV2 conformation changes, exposing ssrA and thus increasing the affinity of the fusion protein to SspB (Lungu et al., 2012). In the absence of light, proteins relax to their ground state. Further engineering of the AsLOV2 domain enabled the creation of a highly efficient system which caused protein dimerization upon light induction and therefore modified the localization or activity of proteins fused to AsLOV2-ssrA and SspB (Guntas et al., 2015; Zimmerman et al., 2016). The affinity of SspB and ssrA in LIDs can be also exploited to regulate assembly of homomeric complexes (Yu et al., 2017). The system was applied in both bacterial and eukaryotic cells (Guntas et al., 2015; Zimmerman et al., 2016; Yu et al., 2017). Fast and reversible action of ssrA-modified LIDs made a good alternative to chemically induced dimerization (Guntas et al., 2015).

### Targeted Protein Degradation Using Degraders

Although fusion proteins with degrons can be used to effectively knock-down proteins in bacteria in a regulated manner, there is still a lack of a universal and adaptable technique which would enable effective degradation of endogenous proteins without any prior modifications with fusion tags. Such approaches have been successfully developed and studied in eukaryotes, which could serve as a starting point for creating analogous techniques for bacteria. We describe the most feasible strategies used in eukaryotes that enable the manipulation of endogenous proteins with the use of exogenously applied compounds.

Targeted protein degradation (TPD) has emerged as a significant technique in drug discovery over the last decade. This approach to treatment omits the limitations of traditionally used inhibitors by elimination of the protein molecules rather than blocking their activity. TPD can also be an alternative to typical reverse genetics methods such as genetic modifications or RNA interference (RNAi) and allows control of protein levels in a fast, precise, and reversible manner. Degradation is triggered by molecules which bring together the protein of interest and the degradation machinery or cause a conformational change of the target which can expose the degron. Degradation-inducing compounds can be small molecules or peptides, and can be a single molecule or a bivalent fusion of two ligands. This technique may lead to significant advances in the treatment of cancer and neurodegenerative diseases which are becoming increasingly prevalent. Three types of TPD agents have shown particular promise so far: PROTACs, molecular glues, and hydrophobic tags.

#### PROTACs

Using Proteolysis-Targeting Chimeras (PROTACs) is a new approach in biological discovery. Typically, a PROTAC is composed of a ligand for a protein of interest joined by a flexible linker to a ligand of an E3 ubiquitin ligase. One advantage of this approach is that PROTACs do not need to occupy an active site, thus they are able to degrade also “classically undruggable” proteins without enzymatic activities such as transcription factors or scaffolding proteins (Gao et al., 2020; Wang et al., 2020). They can also give a new purpose for ligands with a good affinity but poor inhibitory effects or enhance the effects of good inhibitors. The PROTACs themselves are reusable, since after the degradation of one target molecule they can go on to recruit more molecules, which decreases the concentration of the drug required to be effective. Although the design of the molecules appears to be relatively straightforward, there are numerous factors which must be taken into consideration to create an effective PROTAC. Tight binding of the chimeras is achieved by a mechanism of cooperative binding which leads to high ternary affinities. Preferably, the affinity of the PROTAC-target or PROTAC-E3 complexes to the third component (the E3 ligase or the target, respectively) should be higher than the separate binary affinities of the PROTAC components to its individual binding partners (to the E3 ligase or the target) alone (Gadd et al., 2017; Liu X. et al., 2020). Linkers, usually made of PEG or alkyls, play an important role in enabling molecules to form this coordinative and permissive complex by keeping them at a distance which helps to reduce steric constraints but at the same time allows efficient ubiquitination by the proximity effect. The design should take into consideration features such as the length, flexibility, and also the attachment sites of the linker to both ligands, and typically requires optimization for each PROTAC (Cyrus et al., 2011; Maple et al., 2019; Donoghue et al., 2020). The length of the linker not only influences PROTAC action and affinity toward the binding partners but also the compound stability (Goracci et al., 2020; Pike et al., 2020). Another important factor is the cell permeability of PROTAC molecules. The size of a chimera composed of two different ligands is twice as large as traditional drugs, which affects their pharmacokinetics and can potentially cause absorption issues. Surprisingly, PROTAC permeability is relatively high and can be improved by linker modifications or attaching cell-penetrating peptides (Maple et al., 2019; Jin J. et al., 2020; Liu X. et al., 2020). Ligands that bind to the E3 ligase and to the protein of interest can be either small molecules or peptides. The first PROTACs had peptidic binding moieties; however, because of the relatively poor permeability and stability for peptides, more recent PROTACs now are constructed from small molecules (Sakamoto et al., 2001; Schneekloth et al., 2004; Ishikawa et al., 2020). Nonetheless, the limitations of peptide ligands may in principle be obviated with peptidomimetics, chemical modifications, or fusions with cell-penetrating peptides (Jiang et al., 2018; Lu et al., 2018; Au et al., 2020; Jin J. et al., 2020; Lee et al., 2020; Ma D. et al., 2020). Due to the low toxicity of peptides, their large binding surfaces (which can help overcome the effect of mutations in target proteins), and the possibility of designing multiple potential ligands based on structures of protein complexes, peptide-based PROTACs are still used (Au et al., 2020; Jin J. et al., 2020).

**FIGURE 3:**
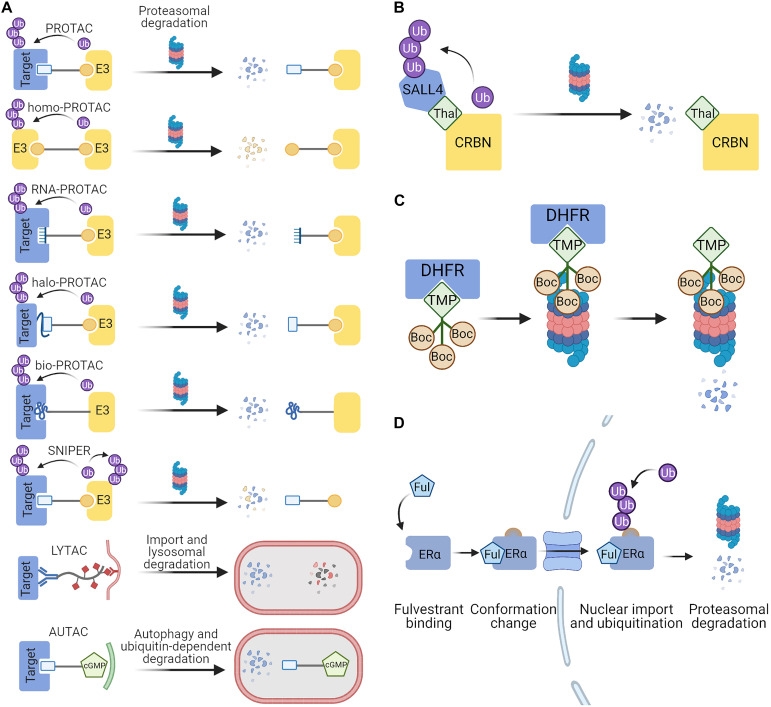
Targeted protein degradation (TPD) strategies exploited in eukaryotes. **(A)** PROTACs are bifunctional chimeras which mediate the recruitment of an E3 ubiquitin ligase to the target protein. PROTAC components can be peptides, small molecules or oligonucleotides recognized as ligands by the target proteins. Ubiquitination of the target results in its degradation by the proteasome, while the PROTAC molecules are recycled for the next proteolytic event. LYTACs and AUTACs direct proteins for lysosomal degradation by promoting their encapsulation in endosomes and autophagosomes, respectively. **(B)** Thalidomide serves as a molecular glue which brings together SALL4 and the cereblon (CRBN) E3 ligase complex. SALL4 becomes a neo-substrate for the ubiquitination by CRBN and is then degraded by the proteasome (Yamanaka et al., 2020). **(C)** Hydrophobic tagging uses chimeric compounds in which a known protein ligand is linked to a highly hydrophobic Boc3-Arg, which is recognized as a degron by the proteasome. DHFR can be targeted for degradation through the use of its ligand trimethoprim in the chimeric hydrophobic tag (Shi et al., 2016). **(D)** Fulvestrant binding to the estrogen receptor α causes conformational changes which exposes the hydrophobic parts of the protein that serve as a degron. The Fulvestrant-bound ERα is degraded in the nucleus through the ubiquitin-proteasome pathway (Cornella-Taracido and Garcia-Echeverria, 2020). Figures were created with BioRender.com.

Even though the most popular PROTACs are minimally made of two peptides or small molecules joined with a linker, a number of modifications to this basic concept have significantly broadened the spectrum of available PROTACs (Figure 3A). This includes light-activated PROTACs (Pfaff et al., 2019; Xue et al., 2019; Jin Y.H. et al., 2020; Liu J. et al., 2020; Manna and Wu, 2020; Reynders et al., 2020), RNA-PROTACs which target RNA-binding proteins (Ghidini et al., 2020), homo-PROTACs which are composed of two particles of the same E3 ligand (Maniaci et al., 2017; Steinebach et al., 2018), HaloPROTACs which are directed against the popular HaloTag (Buckley et al., 2015; Tovell et al., 2019; Simpson et al., 2020), and bioPROTACs composed of E3 ligase fused to known domains that interact with the target protein (Lim et al., 2020). Other techniques which exploit different degradation pathways are Specific and Nongenetic Inhibitor of Apoptosis Protein (IAP)-dependent Protein Erasers (SNIPERs) which have an activity similar to PROTACs but also induce the degradation of the associated ubiquitin ligases (Ohoka et al., 2017; Naito et al., 2019; Ishikawa et al., 2020), LYTACs which degrade extracellular proteins via lysosomal pathway (Banik et al., 2020) or autophagy-inducing AUTACs which can degrade fragmented mitochondria and proteins (Takahashi et al., 2019). All of those approaches create an exciting potential to develop drugs which can target multiple proteins that are untargetable with other methods. Over the last few years there has been a growing interest in PROTACs in both academia and industry as shown by a steep increase in the number of publications on PubMed and patent applications in the Google Patents database. In 2019, the first PROTAC was approved for clinical trials in prostate cancer treatment (Mullard, 2019) and more degraders are soon going to be tested in patients (Mullard, 2021). Most often the chimeric molecules are directed against cancer-related proteins, but are sometimes used in research on neurodegenerative or autoimmune disorders and can even potentially act on viruses such as SARS-CoV2 (Ding et al., 2020; Ocaña and Pandiella, 2020; Tomoshige and Ishikawa, 2020).

#### Molecular Glues

Much like PROTACs, molecular glues are a type of small molecules which brings together two proteins of otherwise poor or no affinity which may lead to a desired outcome such as protein degradation. Molecular glues are typically more compact and less modular than PROTACs, and form a new interface between the two proteins, which results in a high affinity of the ternary complex and less of the pharmacological “hook effect.” Natural examples of such molecules are cyclosporine promoted binding of cyclophilin and calcineurin, and the afore-mentioned rapamycin which acts on FKBP and FRP (Che et al., 2018). In an engineered system using FRP fused to the proteasome and the target protein fused to FKBP, the addition of rapamycin caused ubiquitin-independent proteasomal degradation (Janse et al., 2004). This circumvents the need for an E3 enzyme—a promising premise for the necessarily E3-free TPD in bacteria. Some molecular glues do induce interactions between target proteins and ubiquitin ligases, which causes degradation. For example, a class of anticancer drugs known as SPLAMs cause degradation of RNA-binding protein 39 (RBM39) involved in RNA splicing by the DCAF15 ubiquitin ligase (Che et al., 2018; Faust et al., 2020). Thalidomide derivatives (IMiD) are now known to bind to cereblon (CRBN) E3 ligase complex in the brain and induce degradation of transcription factors such as IZKF1, IZKF3, or SALL4 (Figure 3B). Fusing fragments of those proteins to the protein of interest created a system for IMiD-dependent inducible protein degradation (Koduri et al., 2019; Yamanaka et al., 2020). The discovery of molecular glues has so far been largely serendipitous, albeit once established they often find a widespread use—such as the auxin system derived from plants. Auxin inducible degradation (AID) is used to activate protein degradation in genetically intractable research problems (e.g., studies of cellular memory maintained through epigenetic protein marks; Siwek et al., 2020).

#### Hydrophobic Tagging

A variation on the use of small molecules to induce TPD is a method called hydrophobic tagging. Hydrophobic stretches are often exposed in unfolded proteins, and can be recognized by protein quality control pathways and result in protein degradation (Hachisu et al., 2016). Hydrophobic tags (HyTs) are chimeric compounds designed to have high hydrophobicity and low molecular weight (Neklesa et al., 2011). The primary action of HyTs relies on the recognition of the highly hydrophobic Boc3-Arg (tert-butyl carbamate protected arginine) as the signal for degradation. HyT selectivity is conferred through fusion of Boc3-Arg to a known protein ligand (for example, trimethoprim is used as a ligand of DHFR) (Figure 3C). Alternatively, in the absence of a known ligand, a HaloTag-binding linker can be used to target HaloTag fusion proteins. Degradation mediated by Boc3-Arg is proteasome dependent but ubiquitin independent (Long et al., 2012; Shi et al., 2016). Hydrophobic tagging has been used for inducing the degradation of numerous cancer-related proteins and the Alzheimer disease-related Tau protein (Gao et al., 2017; Rubner et al., 2018, 2019; Nietzold et al., 2019; Ma A. et al., 2020).

#### Small Molecule-Induced Instability

An example of a clinically relevant degradation-promoting small molecule is Fulvestrant, a selective estrogen receptor degrader (SERD), which was approved for breast cancer treatment in 2002 (Bross et al., 2002). It inhibits ER dimerization and its transcriptional activity, and promotes proteasome-dependent degradation (Osborne et al., 2004; Croxtall and McKeage, 2011). It acts by exposing a hydrophobic part of the target ER molecule that mimics a natural degron (Cornella-Taracido and Garcia-Echeverria, 2020) and can be thought of as indirect hydrophobic tagging (Figure 3D). Other small-molecule induced degradation techniques which require engineering the protein of interest include fusions with DHFR or a FKBP12-based destabilizing domain which cover the degrons in the presence of the small molecule ligands but expose them in their absence which results in degradation (Banaszynski et al., 2006; Tai et al., 2012).

#### The First Bacterial Degrader

**FIGURE 4:**
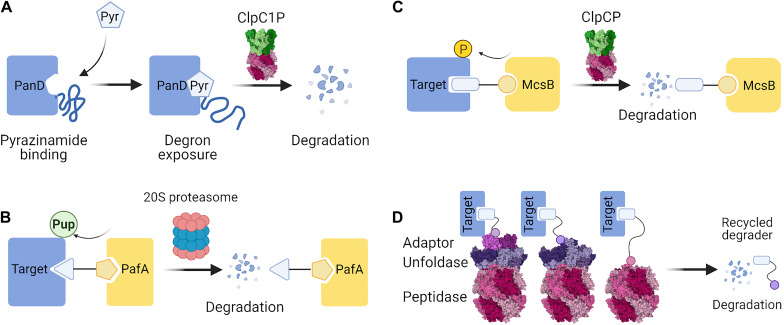
Possible strategies for targeted protein degradation (TPD) in bacteria. **(A)** Pyrazinamide binds PanD, which leads to conformational changes that expose a degron sequence and degradation by ClpC1P (Gopal et al., 2020). **(B)** In Mycobacteria, a PROTAC molecule containing a ligand of PafA could recruit PafA to the target protein. Pupylation of the target protein by PafA could enable its selective degradation by the 20S proteasome. **(C)** In Gram-positive bacteria, the McsB arginine kinase can be exploited to phosphorylate target proteins. A PROTAC containing a ligand of McsB could elicit phosphorylation of the target and bring about ClpCP-mediated degradation. **(D)** PROTAC molecules could directly recruit the proteolytic machinery by employing small molecule or peptide ligands of the proteins involved in the proteolytic pathway. PROTAC-mediated interaction with an adaptor protein, an unfoldase, or a peptidase could serve to induce proximity and cause degradation of the target protein. Figures were created with BioRender.com and Mol* (Sehnal et al., 2018).

Importantly, a recent discovery provided the first example of a small molecule inducing specific protein degradation in bacteria, through induced instability: pyrazinamide. This compound eliminated aspartate 1-decarboxylase PanD activity needed for CoA synthesis in *M. tuberculosis*. It was previously believed to act like a regular inhibitor, but has been recently found to target PanD for degradation by ClpC1P (Gopal and Dick, 2020; Gopal et al., 2020). It acts by exposing the C-terminal degron of PanD and changing the multimeric state of the PanD complex (Figure 4A). This is the first degradation-inducing antimicrobial, working along the lines of SERD-like strategy.

## Discussion

Bacterial proteases are robust machines embedded within tight regulatory networks to ensure timely and specific substrate selection, aided by adaptor proteins and sequence-encoded degradation signals. Their diversity provides researchers with tools for manipulating protein stability in order to investigate protein function and to design useful synthetic circuits. Despite this repertoire, the majority of controlled proteolysis approaches found in the literature and described here focus on variants of ssrA tagging. This might be due to the well-described properties of this system, and its useful modality. Indeed, the applications of ssrA degrons seem versatile and range from large screens of protein function in collections of mutant strains, to elegant reversible switches for *in vivo* studies. It seems that most needs for protein stability control can be addressed using the ssrA degron. However, all of the current approaches to specific and inducible protein degradation in bacteria have one requirement in common: they rely on engineering protein fusions. This might limit their application in terms of the required labor, finding a neutral tagging site, and the genetic engineering tractability of the bacterial species. How would the field progress if the remarkable opportunities offered by PROTACs and molecular glues to target endogenous proteins were also applicable in bacteria?

Several studies employing degrons in bacteria, and the case of pyrazinamide, show that the general requirement for TPD is fulfilled: induced degradation can cause notable molecular and even phenotypic changes despite the typically faster protein turnover rates in bacteria. Moreover, degradation can be brought about simply by virtue of the proximity of the target to the protease, as in the split-adaptor system (Davis et al., 2011). There are, however, few true examples of TPD in bacteria, leaving a significant methodological gap between bacteria and eukaryotes. This stems mostly from the lack of the ubiquitin-proteasome system in bacteria, which has been the foundation for TPD in human cells. Nonetheless, the extensive range of protease action and structures highlighted in this review should enable scientists to ultimately find ways to deliver bacterial proteins of interest for degradation. Here we discuss possible future developments in the light of the present drawbacks and limitations of TPD tools in bacteria.

Firstly, what type of TPD agents may be the most suitable for use in bacteria? While there are various approaches available in eukaryotes, some have a limited potential for becoming the go-to technique for depleting specific endogenous proteins in bacteria. Molecular glues are usually discovered accidentally as they are difficult to rationally design although there were attempts to develop screening techniques enabling identification of potential molecular glues (Mayor-Ruiz et al., 2020). Pyrazinamide is a proof that small-molecule induced instability is a viable strategy (Figure 4A), yet it was also a result of a serendipitous discovery rather than targeted design (Gopal et al., 2020). Similarly, direct hydrophobic tagging of non-fusion proteins has yet to be demonstrated to be a facile tool in bacteria. Some approaches may remain applicable only in eukaryotes, such as those relying on lysosomal degradation. Since we are aiming at engineering a successful and universal strategy, we believe that a PROTAC-like approach would have the most potential to successfully yield bacterial degraders. Similarly to the eukaryotic PROTACs, the bacterial TPD field may start with peptidic degraders and later move on to employing small-molecule chimeras. For example, the multitude of known protein-peptide interactions presents a big repertoire of peptide motifs which could serve as the target-engaging part (warhead) of the bacterial bio-degraders. In addition, based on the success of various eukaryotic PROTACs we hypothesize that it could be possible to create RNA and DNA-degraders which use oligonucleotides as baits for the nucleic acid-binding proteins. By using degraders, it would also be possible to repurpose known small molecules, for example, failed antibiotic candidates which might be decent binders but poor inhibitors. Since only binding (as opposed to any inhibitory potential) is required from the ligand, TPD can bring to bear its key advantage, allowing investigators to target classically “undruggable” proteins without any tractable active sites.

What seems to be a more challenging task is finding an effective method for delivering the proteins of interest to the proteases. Because of the lack of ubiquitin-dependent degradation, it is necessary to find a different strategy to recruit proteolytic machinery to the target. One of the possible solutions would be recruitment of the PafA Pup-ligase which could result in pupylation and targeting the protein to the bacterial proteasome (Figure 4B). This approach would be applicable in a limited number of bacteria, although it could help to create new tools and antibiotics against *Mycobacterium tuberculosis*. Since proteins phosphorylated on arginine residues are known substrates of ClpCP, recruitment of the McsB kinase is also a promising TPD strategy (Suskiewicz et al., 2019) in Gram-positive bacteria (Figure 4C). In a more universal approach, bacterial degraders could directly recruit a proteolytic complex without relying on a post-translational modification step. The viability of this strategy is hinted at by the studies successfully employing rapamycin-mediated interaction with the target to bring about proteasomal (Janse et al., 2004) or ClpXP-mediated (Davis et al., 2011) degradation. Bacterial degraders could recruit proteolytic activity by employing a ligand binding to an adaptor protein, an unfoldase, or even the protease component (Figure 4D) from the suggested repertoire described above (Table 1). Finally, future work may find ways to exploit other pathways unique to bacteria, for example by promoting trans-translation to append ssrA or poly-Ala tails in a target-specific manner, although currently such precise action cannot be yet achieved.

If the bacterial degraders have to rely on direct protease recruitment, how would their characteristics compare with those of eukaryotic PROTACs? The first concern is that peptidic degraders may be degraded together with their targets, losing the potential to be recycled and to gain a catalytic-like efficiency of their eukaryotic counterparts. Peptide mimics or switching to small-molecule ligands may be required to ensure the stability of the degraders. In general, promoting ternary (i.e., target-degrader-E3 ligase) complex formation is a key concern in TPD, and in this aspect molecular glues are better candidates than PROTACs. Similarly, in bacterial TPD the best compounds would promote target interaction with a part of the protease complex that engages substrates. This requires careful optimization of PROTAC linkers in terms of distance, flexibility, and promoted stereochemistry. For the majority of the eukaryotic degraders, the rate-limiting step seems to be enzymatic reaction initiation (monoubiquitin transfer) right after the ternary complex formation, since it requires spatial alignment of the active site and the target Lys residue (Fisher and Phillips, 2018). In bacteria, the equivalent rate-limiting step might be the substrate engagement in the unfoldase or protease pore; once initiated, the motor action of the ATPase might help in further progress of the proteolysis. Unlike PROTACs engaging a novel E3 ligase, proteins targeted for degradation in bacteria may not necessarily be neo-substrates for the recruited proteases. Naturally occurring, less structured sites and loops may help in achieving substrate unfolding for proteolysis, while preferably slow dissociation rates would help bacterial degraders potentiate this initiation event. On the other hand, degraders binding too tightly to their targets might preclude efficient proteolysis by stabilizing or sequestering the target from the reach of the protease. An adaptor-recruiting degrader which binds too tightly to the protease adaptor might also cause degradation of the adaptor following the engagement of the target. In such cases, the affinity of the degrader would need to be fine-tuned in order to bind sufficiently strongly to selectively bring the target to the proteolytic complex, yet loosely enough to allow extraction upon engagement and to release the target upon the proteolytic event. It may be possible to find a way to rescue adaptors and degraders, similarly to the natural resistance of certain adaptors that bind and even bait the unfoldases yet avoid destruction along with the substrate. This may be conferred by conformational changes associated with substrate unfolding and degradation, though the details of such mechanisms are still poorly understood and are not easily engineered. Finally, the problem of delivering the degraders into the bacterial cells would need to be addressed. Possibly, some modifications could be added to promote the active import of degraders which would obviate the issues with the larger sizes of chimeric compounds (e.g., conjugates with Proline rich AntiMicrobial Peptides (PrAMPs) (André et al., 2020) may help degraders enter the cell). It will be exciting to see how all of these concerns will be addressed by the first true TPD studies in the future.

Once these challenges are overcome, bacterial degraders could provide an excellent alternative reverse-genetics approach for studying protein function, opening new possibilities such as dose-dependent and time-resolved control that would supersede the use of gene knockouts and protein fusions. The unique suitability of TPD for studying fast biological processes may be especially appreciated for applications in bacteria, whose molecules typically have shorter half-lives due to the higher metabolic rates compared to human cells. More importantly, degraders could also constitute a completely novel and possibly resistance-retardant class of antibiotics, which gains importance in the light of increasing antimicrobial resistance (O’Neill, 2016). The recent COVID-19 outbreak proves that infectious diseases are still a global threat, and excessive use of antibiotics during the pandemic has exacerbated the growth of antimicrobial resistance even further (Arshad et al., 2020). Therefore, degrader-type antibiotics could be of particular interest, since the antiviral PROTACs have been shown to act fast enough to prevent the rise of viral resistance (de Wispelaere et al., 2019). The current state of the art is ripe for the design and exploration of TPD in bacteria, and the expected results will open a plethora of opportunities both for research and in antimicrobial therapies.

## Author Contributions

MI wrote the first draft of the manuscript. MG wrote sections of the manuscript. All authors edited and approved the submitted version, contributed to the conception and preparation of the manuscript.

## Conflict of Interest

The authors declare that the research was conducted in the absence of any commercial or financial relationships that could be construed as a potential conflict of interest.
